# 

**Published:** 1982

**Authors:** 


### Contents

3 Editorial

4 Presidential Address,
Medicine and the Bristol Zoo
Robert Warin

12 Fungi, Food, Poison and Mystery

Philip Radford

16 A Literary Evening at the Watershed Centre
with Oliver Neville and Pat Heywood

25 From our Correspondents

28 West Country Physicians Club,
Spring Meeting 1982

31 Surgical Club of South West England,
Spring Meeting 1982

37 Southmead Centre for Medical Education

38 Cossham Medical Society

39 British Medical Association, Bristol Division

40 Programmes of Societies, 1982–83
Cossham Medical Society
Bristol Medic-Legal Society

41 Examination Results

